# Assessing circulating tumour DNA (ctDNA) as a prognostic biomarker in locally advanced rectal cancer: a systematic review and meta-analysis

**DOI:** 10.1007/s00384-024-04656-1

**Published:** 2024-05-29

**Authors:** Niall J. O’Sullivan, Hugo C. Temperley, Eimear T. Kyle, Kevin J. Sweeney, Maeve O’Neill, Charles Gilham, Jacintha O’Sullivan, Grainne O’Kane, Brian Mehigan, Sharon O’Toole, John Larkin, David Gallagher, Paul McCormick, Michael E. Kelly

**Affiliations:** 1 Department of Surgery, St. James’s Hospital, Dublin 8, Ireland; 2 School of Medicine, Trinity College Dublin, Dublin 2, Ireland; 3 Department of Radiation Oncology, St. James’s Hospital, Dublin 8, Ireland; 4 Trinity Translational Medicine Institute, Trinity St. James’s Cancer Institute, Trinity College, St. James’s Hospital, Dublin, Ireland; 5 Department of Medical Oncology, St. James’s Hospital, Dublin 8, Ireland; 6 Department of Genetics, St. James’s Hospital, Dublin 8, Ireland; 7 Trinity St. James’s Cancer Institute, St. James’s Hospital, Dublin 8, Ireland

**Keywords:** Rectal cancer, ctDNA, Recurrence, Survival

## Abstract

**Introduction:**

Circulating tumour DNA (ctDNA) has emerged as a promising biomarker in various cancer types, including locally advanced rectal cancer (LARC), offering potential insights into disease progression, treatment response and recurrence. This review aims to comprehensively evaluate the utility of ctDNA as a prognostic biomarker in LARC.

**Methods:**

PubMed, EMBASE and Web of Science were searched as part of our review. Studies investigating the utility of ctDNA in locally advanced rectal cancer (LARC) were assessed for eligibility. Quality assessment of included studies was performed using the Newcastle Ottawa Scale (NOS) risk of bias tool. Outcomes extracted included basic participant characteristics, ctDNA details and survival data. A meta-analysis was performed on eligible studies to determine pooled recurrence-free survival (RFS).

**Results:**

Twenty-two studies involving 1676 participants were included in our analysis. Methodological quality categorised by the Newcastle Ottawa Scale was generally satisfactory across included studies. ctDNA detected at various time intervals was generally associated with poor outcomes across included studies. Meta-analysis demonstrated a pooled hazard ratio of 8.87 (95% CI 4.91–16.03) and 15.15 (95% CI 8.21–27.95), indicating an increased risk of recurrence with ctDNA positivity in the post-neoadjuvant and post-operative periods respectively.

**Conclusion:**

Our systematic review provides evidence supporting the prognostic utility of ctDNA in patients with LARC, particularly in identifying patients at higher risk of disease recurrence in the post-neoadjuvant and post-operative periods.

**Supplementary Information:**

The online version contains supplementary material available at 10.1007/s00384-024-04656-1.

## Introduction

Locally advanced rectal cancer (LARC) can cause significant challenges in terms of management [1]. Increasingly, patients are having total neoadjuvant treatment (TnT) [2], and the need for better indicators of complete clinical response, especially in borderline cases, is vital [3]. Circulating tumour DNA (ctDNA) has emerged as a promising biomarker in various cancer types, including LARC, offering potential insights into disease progression, treatment response and recurrence [4–8].

ctDNA refers to fragmented DNA shed by tumour cells into the bloodstream [9]. These fragments carry genetic alterations characteristic of the originating tumour, providing a non-invasive means of interrogating tumour biology [10]. The detection and analysis of ctDNA have garnered significant interest in cancer research due to its potential applications in diagnosis, prognostication and treatment monitoring [11]. ctDNA can be isolated from peripheral blood samples and analysed using various techniques, including next-generation sequencing (NGS), digital PCR (dPCR) and targeted amplicon-based assays [12].

NGS is a highly sensitive technique that allows for the comprehensive profiling of ctDNA, enabling the detection of a wide range of genetic alterations, including single nucleotide variants (SNVs), insertions and deletions (indels), copy number variations (CNVs) and structural rearrangements [13]. Conversely, dPCR quantifies the absolute number of target DNA molecules, allowing for precise measurement of ctDNA levels and increased cost-effectiveness when compared to NGS [14]. Alternative approaches include real-time PCR (qPCR), BEAMing and fragment analysis [15–17]. By profiling the genomic landscape of tumours through ctDNA analysis, clinicians can gain insights into tumour heterogeneity, clonal evolution and potential therapeutic targets, thereby facilitating personalised treatment approaches [18].

While several clinicopathological factors (MRI and endoscopic response) are currently used to stratify response to treatments in patients with LARC, they have limitations [19]. In addition, traditional prognostic factors such as tumour stage and histological grade can provide circumstantial value about potential disease behaviour and treatment response. There is a need to identify novel biomarkers that can complement existing prognostic tools and enhance risk stratification in LARC [20]. This systematic review aims to comprehensively evaluate the utility of ctDNA as a prognostic biomarker in LARC, exploring its potential to address the unmet clinical needs in this challenging disease context.

## Methods

### Study design and reporting guidelines

This is a systematic review and meta-analysis of retrospective and prospective cohort studies conducted in accordance with the Preferred Reporting Items for Systematic Reviews and Meta-Analyses (PRISMA) reporting guidelines [21]. Local institutional ethical approval was not required. All authors declare no conflicts of interest. This research received no external funding.

### Search strategy

The following databases were searched as part of our systematic review process in April 2024; MEDLINE, PubMed, Embase and Web of Science. The following search strategy was used: (rectal OR rectum OR colorect*) AND (circulating tumour DNA OR ctDNA OR ct-DNA OR circulating free DNA OR cfDNA or cf-DNA). The search was completed on 5th April 2024. The grey literature was also searched as part of our study to identify any further ongoing works of literature, including theses and conference abstracts.

### Eligibility criteria

Original studies investigating an association between ctDNA and treatment or oncological outcomes in patients with locally advanced rectal cancer were eligible for inclusion. Case reports, conference abstracts and review articles were excluded.

### Data extraction and quality assessment

A database was established utilising the citation management software EndNote X9TM. Independent reviews of search outputs were conducted by two researchers (NOS and HCT). Initially, duplicate entries were eradicated, followed by a screening of study titles to gauge potential relevance. Subsequently, the abstracts of selected studies underwent assessment for eligibility based on predetermined inclusion/exclusion criteria. Excluded studies were categorised by reason within the database. Full texts of eligible abstracts were then scrutinised using identical criteria.

For efficient data extraction and storage, the Cochrane Collaboration’s screening and data extraction tool, Covidence, was employed [22]. Data collection was undertaken independently by two reviewers (NOS and HCT), encompassing study details, design, population, intervention, comparison groups and outcomes. Discrepancies between reviewers were resolved through open discussion, with final arbitration by the senior author (MK).

Potential biases in non-RCT studies were evaluated using the Newcastle-Ottawa Scale (NOS) risk of bias tool, with results tabulated accordingly [23]. This tool assesses studies across various categories, assigning stars to denote quality: 7 stars indicating “very good,” 5-6 stars for “good,” 3-4 stars for “satisfactory,” and 0–2 stars for “unsatisfactory.” Two reviewers (NOS and HCT) independently conducted critical appraisals, with a third reviewer (MK) arbitrating in cases of discordance.

### Statistical analysis

Statistical analysis was conducted using Revman Statistical Software (Ver. 5 Copenhagen, Denmark). Generic inverse variance data were presented as hazard ratios (HR) alongside 95% confidence intervals (95% CI). Outcome measures, including mean with standard deviation and median with interquartile range, were documented. Only studies that provided hazard ratios with either confidence intervals or *p*-values were eligible for inclusion in the meta-analysis. When necessary, outcome variables (mean and SD) were estimated from the median and range using the formula described by Hozo et al. [24]. Heterogeneity was evaluated using *I*-squared statistics, with values exceeding 50% indicating significant heterogeneity. Statistical significance was defined as a *p*-value of less than 0.05. A random effects model was employed uniformly.

### Systematic review registration

Our systematic review was registered on PROSPERO in April 2024 (ID: 533712).

## Results

### Search results

The previously outlined literature search yielded a total of 2123 results (Supplementary material S1). After eliminating 281 duplicates, 1842 studies underwent screening. Following the initial screening, 73 full abstracts were meticulously reviewed for eligibility, resulting in 42 being selected for full-text scrutiny. Among these 42 full texts, 22 studies met the eligibility criteria and were consequently included in our analysis [25–46]. Notably, eight of the included studies provided adequate statistical data for incorporation into our quantitative analysis [30, 32–34, 36, 42, 44, 45]. A detailed depiction of the literature screening process can be found in Supplementary material S1.

### Methodological characteristics and quality of studies

Among the 22 studies included, seventeen were conducted prospectively, four retrospectively, and one did not specify the study design. Regarding the study settings, ten were conducted in a single institute, nine in a multi-institutional setting, and three did not specify. Concerning the assessment of the risk of bias, eleven studies were rated as “very good”, ten as “good”, one as “satisfactory” and none as “unsatisfactory” according to the classification by the Newcastle Ottawa Scale. Supplementary material S2 provides a summary of our risk of bias assessment. The methodological characteristics of the included studies are outlined in Supplementary material S3.

### Participant characteristics

The total number of participants across the included studies was 1676. All patients had a diagnosis of locally advanced rectal cancer. Baseline participant characteristics are outlined below in Table 1.

**Table 1:** Baseline participant characteristics

**Name**	**#Patients**	**Median age (range)**	**Male to female**	**Tumour class**	**Assay type**
Agostini 2011	67	61 (20–79)	42:25	LARC	cfDNA concentration
Alden 2024	44	56 (32080)	26:18	LARC	Mutation-specific panel
Appelt 2020	146	64 (57–69)	93:53	LARC	cfDNA concentration
Boysen 2017	75	68 (35–85)	45:30	LARC	cfDNA concentration
Guo 2020	194	n/a	n/a	LARC	Promoter genes
Hofste 2023	51	66 (48–84)	37:14	LARC	Mutation-specific panel
Khakoo 2019	47	59 (30–83)	29:18	LARC	Mutation-specific panel
Liu 2022	60	n/a	n/a	LARC	Mutation-specific panel
McDuff 2021	29	54 (48–66)	15:14	LARC	Mutation-specific panel
Morais 2023	18	65 (38–86)	8:10	LARC	Mutation-specific panel
Murahashi 2020	85	60 (52–69)	65:20	LARC	Mutation-specific panel
Pazdirek 2020	36	64.1	27:9	LARC	Mutation-specific panel
Roesel 2022	25	65 (59–70)	22:3	LARC	Mutation-specific panel
Schou 2017	123	67	74:49	LARC	cfDNA concentration
Sclafani 2018	97	59	36:23	LARC	Mutation-specific panel
Sun 2014	34	57 (29–73)	19:15	LARC	Multiple
Tie 2018	159	62	107:52	LARC	Mutation-specific panel
Truelsen 2022	76	68 (25–94)	41:35	LARC	cfDNA concentration
Vidal 2021	62	62 (33–75)	40:22	LARC	Mutation-specific panel
Wang 2021	119	57	85:34	LARC	Mutation-specific panel
Zhou 2020	104	60 (26–74)	67:37	LARC	Mutation-specific panel
Zitt 2008	25	64 (34–83)	18:7	LARC	cfDNA concentration

*LARC* locally advanced rectal cancer

### ctDNA details and study findings

ctDNA assay type, sequencing method and collection time points varied across included studies. Fourteen studies measured a mutation-specific panel, six measured cfDNA concentration and the remaining two measured promoter genes or multiple assays respectively. In regard to the sequencing method, next-generation sequencing (NGS) was the most commonly utilised method accounting for ten studies, followed by polymerase chain reaction (PCR) (*n* = 9) and direct fluorescent antibody (DFA) (*n* = 2). One study failed to report the sequencing method.

In terms of approach, eleven studies used an agnostic, ten used targeted and one study used both approaches. Agnostic approaches do not rely on prior knowledge of specific mutations but instead analyse the entire ctDNA for any alterations that may be present [47]. These approaches are valuable in settings where comprehensive genomic profiling is necessary, particularly for identifying novel or unexpected mutations. They are beneficial in research and clinical scenarios where a wide array of genetic alterations needs to be detected to inform treatment decisions or understand tumour heterogeneity [48]. Conversely, targeted approaches focus on detecting specific, known mutations that are of clinical interest [49]. They often use panels designed to target commonly mutated genes in particular cancers. Targeted approaches are highly relevant in clinical settings where specific genetic alterations are known to influence prognosis or guide targeted therapies [50]. They are typically more cost-effective and faster than agnostic approaches, making them suitable for routine clinical use, particularly when monitoring known mutations for treatment response or minimal residual disease. Both approaches have their place in the evaluation of ctDNA [47]. Agnostic methods provide a broad view of the genetic landscape, which can uncover new targets for therapy, while targeted methods allow for focused, efficient and cost-effective monitoring of known genetic alterations.

Tables 2 and 3 below outline ctDNA panel details, collection timepoints and main study findings.

**Table 2:** ctDNA details

**Name**	**Assay type**	**Sequencing method**	**Tumour informed**	**Collection timepoints**
Agostini 2011	cfDNA concentration	PCR	Agnostic	Pre-CRT, Post-CRT
Alden 2024	Mutation-specific panel	n/a	Tumour informed	Pre-CRT, Post-CRT
Appelt 2020	cfDNA concentration	PCR	Agnostic	BL
Boysen 2017	cfDNA concentration	PCR	Agnostic	Post-CRT
Guo 2020	Promoter genes	NGS	Agnostic	BL
Hofste 2023	Mutation-specific panel	NGS	Tumour informed	BL, post-CRT, post-op
Khakoo 2019	Mutation-specific panel	PCR	Tumour informed	BL, intra-CRT, post-CRT, post-op
Liu 2022	Mutation-specific panel	NGS	Both	Intra-NAT, post-NAT
McDuff 2021	Mutation-specific panel	PCR	Tumour informed	BL, pre-op, post-op
Morais 2023	Mutation-specific panel	NGS	Agnostic	BL ± post-CRT
Murahashi 2020	Mutation-specific panel	NGS	Agnostic	BL, post-NAT, post-op
Pazdirek 2020	Mutation-specific panel	PCR	Tumour informed	BL, during CRTx
Roesel 2022	Mutation-specific panel	NGS	Tumour informed	T0: day 1 RTx Tend: last day RTx T4: 4/52 post RTx T7: 7/52 post RTx Top: day of surgery, Tpost-op: 3–7/7 post-op TIMV: mesenteric vein sampling during surgery
Schou 2017	cfDNA concentration	dFA	Agnostic	BL, post-induction CTx, post-CRT, serial samples 5 years post-op
Sclafani 2018	Mutation-specific panel	PCR	Tumour informed	BL
Sun 2014	Multiple	PCR	Agnostic	BL, post-CRT
Tie 2018	Mutation-specific panel	NGS	Tumour informed	BL, post-CRT, post-operative
Truelsen 2022	cfDNA concentration	dFA	Agnostic	BL, mid and post-therapy
Vidal 2021	Mutation-specific panel	NGS	Agnostic	BL, during CRT, post-op
Wang 2021	Mutation-specific panel	NGS	Tumour informed	BL, post-NAT
Zhou 2020	Mutation-specific panel	NGS	tumour informed	BL, during CRT, pre- and post-op
Zitt 2008	cfDNA concentration	PCR	Agnostic	BL, post-CRT, end treatment

*cfDNA* circulating-free DNA, *PCR* polymerase-chain reaction, *NGS* next-generation sequencing, *CRT* chemoradiotherapy, *BL* baseline, *Post-op* post-operative, *NAT* neoadjuvant therapy, *CTx* chemotherapy, *RTx* radiotherapy, *dFA* direct fluorescent assay

**Table 3:** Study findings

**Name**	**1° Outcome**	**Main finding**	**Statistical analysis**
Agostini 2011	pCR	Post-CRT levels of cfDNA integrity index significantly lower in responsive compared to non-responsive disease	
Alden 2024	cCR + pCR	Post-TNT ctDNA had sensitivity 23% and specificity 100% for predicting residual disease post-resection	
Appelt 2020	5-year OS, distant metastases	Presence of meth-ctDNA was a/w a worse 5-year OS and rate of distant metastasis	5-year OS: 47% vs 69%, HR 2.08, 95% CI (1.23–1.51) 5-year distant mets: 55% vs 72%, HR 2.20, 95% CI (1.19–4.07)
Boysen 2017	Stage and recurrence	Increased cfDNA a/w higher AJCC stage and recurrence	
Guo 2020	pCR	PPCET gene panel predicted pCR with good performance	AUC 0.89 (0.83–0.94), sensitivity 0.85, specificity 0.93
Hofste 2023	Recurrence	ctDNA detection post-CRT and post-operatively a/w worse progression-free survival	Post-CRT: DFS: HR 6.5, 95% CI (1.4–30.3), *p* = 0.02) Post-op DFS: HR 10.9, 95% CI (1.1–106.7), *p* = 0.04
Khakoo 2019	mrTRG, metastasis-free survival,	Metastases-free survival shorter in patients with detectable ctDNA post-CRT, pre + mid CRT and pre + mid + after CRT	MFS post-CRT: HR 7.1, 95% CI (2.4–21.5) MFS pre + mid-CRT: HR 3.8, CI (1.2–11.7) MFS pre + mid + post-CRT: HR 11.5, 95% CI (3.3–40.4)
Liu 2022	Recurrence	Positive minimal residual disease (MRD) based on post-NAT personalised assay a/w increased risk of recurrence	Recurrence: HR 27.38, 95%CI (8.61–87.06)
McDuff 2021	Recurrence-free survival	Detectable post-op ctDNA a/w poor RFS	(HR 11.56, 95% CI 1.95–68.47 *p* 0.007)
Morais 2023	Disease-free survival, response to treatment	ctDNA a/w poor response to treatment, patients with 2 or more mutations had worse DFS (*p* = 0.005)	
Murahashi 2020	pCR, recurrence	High post-operative ctDNA a/w poor recurrence-free survival	HR 7.7 (95% CI 1.6–42)
Pazdirek 2020	Disease-free survival	Pre-treatment ctDNA presence a/w reduced post-operative DFS and OS	
Roesel 2022	pCR	Day of surgery positive ctDNA a/w poor response to NACRT	
Schou 2017	Recurrence-free survival, DFS	High BL cfDNA levels a/w shorter RFS and DFS	RFS: HR 2.48, 95%CI (1.3–4.8) DFS: HR 2.43, 95% CI (1.27–4.7)
Sclafani 2018	Treatment response	KRAS mutation detection in ctDNA failed to predict prognosis	
Sun 2014	TRG	Higher MGMT promoter methylation status at BL DNA a/w better tumour response	
Tie 2018	Recurrence, RFS	ctDNA detection post-CRT or post-op a/w worse RFS + predictive of recurrence	Post-CRT RFS: HR 6.6 CI 2.6–17, post-op RFS: HR 13.0 CI 5.5–31
Truelsen 2022	pCR	Low post-tx cfDNA + 'cfDNA responders' a/w improved rate pCR	
Vidal 2021	pCR, recurrence, DFS, OS	ctDNA status not a/w pCR. Post-op ctDNA presence a/w recurrence, shorter DFS and shorter OS	RFS: HR 4.029, 95% CI (1.004–16.16) OS: HR 23, 95% CI (2.4–212)
Wang 2021	pCR, RFS	ctDNA clearance a/w low probability of non-pCR. CRC driver gene in ctDNA detection post-CRT a/w worse RFS and recurrence	RFS: HR 9.29, 95% CI (3.74–23.10) Recurrence: HR 90.29, 95% CI (17.01–479.26)
Zhou 2020	pCR, RFS	Low pre-op ctDNA a/w good pCR. ctDNA positivity at all timepoints a/w shorter metastasis-free survival	
Zitt 2008	Treatment response	Decreased post-tx ctDNA a/w treatment response	

*pCR* pathological complete response, *cCR* clinical complete response, *CRT* chemoradiotherapy, *cfDNA* circulating free DNA, *TNT* total neoadjuvant therapy, *OS* overall survival, *HR* hazard ratio, *CI* confidence interval, *AJCC* American Joint Committee on Cancer, *MFS* metastasis-free survival, *a/w* associated with, *DFS* disease-free survival, *RFS* recurrence-free survival, *TRG* tumour regression grade, *NACRT* neoadjuvant chemoradiotherapy, *mrTRG* magnetic-resonance TRG

### Meta-analysis

**Fig. 1:**
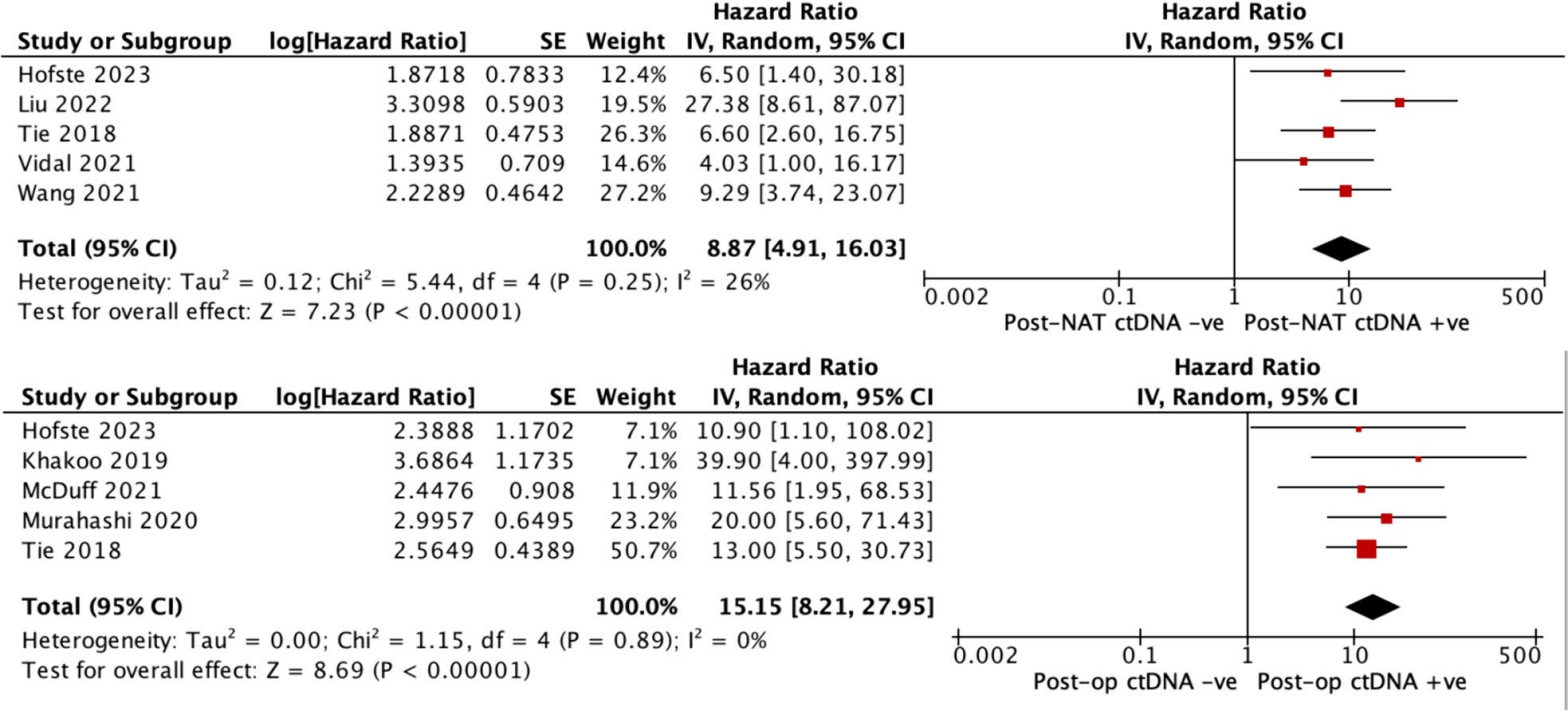
Top: post-NAT ctDNA meta-analysis. Bottom: post-operative ctDNA meta-analysis

Eight of the included studies provided sufficient statistical data to be included in our quantitative analysis [30, 32–34, 36, 42, 44, 45]. We investigated for an association between recurrence-free survival and the presence of ctDNA at several timepoints in a meta-analysis, the results of which are demonstrated in Fig. 1 below. The pooled hazard ratio for ctDNA presence after completion of neoadjuvant therapy when compared to patients who were not found to have detectable ctDNA was 8.87 (95% CI 4.91–16.03, *p* = < 0.0001)). Similarly, the pooled hazard ratio for ctDNA presence post-operatively compared to those without detectable ctDNA was 15.15 (95% CI 8.21–27.95, *p* = < 0.0001)). These results indicate an increased risk for recurrence in patients with LARC with detectable ctDNA in either the post-neoadjuvant therapy or post-operative periods.

### Ongoing research

Several trials are ongoing to further determine the prognostic capabilities of ctDNA in patients with LARC. The DYNAMIC-RECTAL trial (ACTRN12617001560381) is a multi-centre randomised controlled phase 2 trial aiming to investigate the prognostic benefits of ctDNA detection post-operatively to guide the need for adjuvant treatment in patients with LARC. Eligible patients underwent neoadjuvant chemoradiation and total mesenteric excision, and the primary endpoint was adjuvant chemotherapy use. Preliminary results suggest that fewer patients in the ctDNA-guided arm required adjuvant therapy with a lower risk of recurrence in patients with undetectable post-operative ctDNA [51].

The SYNCOPE study (NCT04842006) is a randomised controlled treatment trial aiming to reduce both over-treatment and metastatic disease progression in patients with rectal cancer. Participants with high-risk features will be randomised into two groups: early systemic chemotherapy followed by ctDNA and organoid-guided adjuvant therapy, or conventional treatment. Primary outcomes include RFS and ctDNA positivity rates in post-operative patients within the conventional treatment arm not exposed to chemotherapy.

The REVEAL study (NCT05674422) is a prospective multi-institutional study evaluating response to total neoadjuvant therapy (TNT) with liquid biopsy in eligible patients with LARC. The study aims to investigate the role of ctDNA in the prediction of relapse in this cohort of patients followed by a watch and wait programme or TME depending on the response to initial treatment.

Finally, CINTS-R (NCT05601505) is a multi-institutional randomised controlled trial aiming to evaluate outcomes of ctDNA-guided neoadjuvant treatment in patients with LARC. Treatment groups will receive either NCRT followed by immunotherapy, NCRT alone or TNT depending on mutation status following NGS of tumour tissue and variant allele frequency (VAF) of ctDNA. Control groups will receive standard NCRT only.

## Discussion

Our systematic review of the current available literature demonstrates the significant potential of ctDNA as a prognostic biomarker in patients with LARC. Particularly, ctDNA detection post-neoadjuvant therapy or post-operatively was associated with an increased risk of recurrence, suggesting its utility in predicting disease progression and informing treatment decisions. Meta-analysis of available data further supported these findings, indicating a significantly higher hazard ratio for recurrence in patients with detectable ctDNA at these critical time points. These results align and extend upon existing literature on ctDNA in various cancer types, highlighting its potential as a non-invasive biomarker for monitoring disease burden and treatment response in a multitude of malignancies [4–8].

Despite its promise, ctDNA analysis is not without limitations [52]. Technical challenges, such as low concentrations of ctDNA in circulation and the need for sensitive detection methods, can impede accurate assessment. Similarly, tumour heterogeneity and clonal evolution may further complicate interpretation, potentially leading to false-negative or false-positive results [53]. Moreover, the lack of standardised protocols for ctDNA analysis and variability in assay performance across studies underscore the need for methodological standardisation and validation [54].

While our systematic review provides valuable insights into the role of ctDNA in LARC, several limitations warrant consideration. Firstly, studies included in our review demonstrated heterogeneity in terms of methodology, patient populations and outcome measures, which may have introduced bias and confounded interpretation. Additionally, the reliance on published data may have led to publication bias, with studies reporting significant findings more likely to be included. Despite these limitations, the findings of our review have significant implications for future research and clinical practice. The consistent association between ctDNA detection and adverse outcomes in LARC suggests its potential as a prognostic biomarker for risk stratification and treatment decision-making. Integrating ctDNA analysis into routine clinical practice could facilitate personalised treatment strategies, including the identification of high-risk patients who may benefit from intensified therapy or closer surveillance [55]. Furthermore, ongoing research efforts aimed at refining ctDNA-based assays and elucidating the underlying biological mechanisms driving ctDNA release and clearance are crucial for maximising its clinical utility [56].

Future research in this field should prioritise several key areas to address current knowledge gaps and optimise the implementation of ctDNA analysis in clinical practice. Firstly, large-scale prospective studies with standardised methodologies are needed to validate the prognostic utility of ctDNA across diverse patient populations and treatment settings [57]. Additionally, efforts to standardise ctDNA assays, including sample collection, processing and analysis protocols, are essential to ensure the reproducibility and comparability of results. Finally, collaborative efforts to establish international consortia and biobanks for ctDNA research could facilitate data sharing and accelerate progress towards clinical implementation [58].

## Conclusion

Our systematic review provides evidence supporting the prognostic utility of ctDNA in patients with LARC, particularly in identifying patients at higher risk of disease recurrence in the post-neoadjuvant and post-operative periods.

## Supplementary Information

Below is the link to the electronic supplementary material.

Supplementary file1 (DOCX 49 KB)

## Data Availability

No datasets were generated or analysed during the current study.
