# Supplementary material for: Assessing circulating tumour DNA (ctDNA) as a prognostic biomarker in locally advanced rectal cancer: a systematic review and meta-analysis
Source: Int J Colorectal Dis. 2024 May 29;39(1):82. doi: 10.1007/s00384-024-04656-1 (PMC11136793; doi:10.1007/s00384-024-04656-1)
Supplement: Supplementary file 1 — Supplementary file1 (DOCX 49 KB) [file 384_2024_4656_MOESM1_ESM.docx]

**Supplementary material S1: Study selection.** A PRISMA Flowchart of the selection of relevant publications included in this review

**Records identified through database search**
(n = 2123)

**Records excluded**
(n = 1800)

Deleted based on title = 1444

Deleted based on abstract = 356

**Full-text articles assessed for eligibility**
(n = 42)

**Records after duplicates removed**
(n = 1842)

**Records screened**
(n = 1842)

Identification

Screening

**Full-text articles excluded with reasons**
(n = 20)

**Study Design = 11**

*Conference abstracts (6)*

*Case reports (5)*

**Population = 5**

*Colon cancer (5)*

**Intervention = 3**

*No ctDNA analysis (3)*

**Outcome = 1**

*No measurable outcomes (1)*

Eligibility

**Studies included in qualitative synthesis**
(n = 22)

Included

**Studies included in quantitative synthesis**
(n = 8)

**S2: Risk of bias assessment (Newcastle-Ottawa scale)**

| Author |  | **Selection** | | | | **Comparability.** | **Outcome** | | | **Quality** |
| --- | --- | --- | --- | --- | --- | --- | --- | --- | --- | --- |
|  |  | **Representativeness of the exposed cohort** | **Sample size (<20 = no star)** | **Open cases only included** | **Ascertainment of the exposure** | **The subjects in different outcome groups are comparable** | **Assessment of outcome** | **Less than 10% missing data?** | **Average Follow up period (> 12 months)** |  |
| Agostini | 2011 | **🟑** | **🟑** | **🟑** | **🟑** | / | **🟑** | **🟑** | / | 6 |
| Alden | 2024 | **🟑** | **🟑 ** | **🟑** | / | **🟑** | **🟑** | **🟑** | **🟑** | 7 |
| Appelt | 2020 | **🟑** | **🟑** | **🟑** | **🟑** | **🟑** | **🟑** | / | / | 6 |
| Boysen | 2017 | **🟑** | **🟑** | / | **🟑** | **🟑** | / | **🟑** | / | 5 |
| Guo | 2020 | **🟑** | **🟑** | **🟑** | **🟑** | **🟑** | **🟑** | **🟑** | **🟑** | 8 |
| Hofste | 2023 | **🟑** | **🟑 ** | **🟑** | **🟑** | **🟑** | **🟑** | / | **🟑** | 7 |
| Khakoo | 2019 | **🟑** | **🟑** | **🟑** | **🟑** | **🟑** | **🟑** | **🟑** | **🟑** | 8 |
| Liu | 2022 | **🟑** | **🟑** | **🟑** | **🟑** | **🟑** | **🟑** | **🟑** | **🟑** | 8 |
| McDuff | 2021 | **🟑** | / | / | / | **🟑** | / | **🟑** | **🟑** | 4 |
| Morais | 2023 | **🟑** | **🟑** | **🟑** | / | **🟑** | **🟑** | **🟑** | / | 6 |
| Murahashi | 2020 | **🟑** | **🟑** | **🟑** | **🟑** | / | **🟑** | **🟑** | **🟑** | 6 |
| Pazdirek | 2020 | **🟑** | **🟑 ** | **🟑** | **🟑** | **🟑** | / | **🟑** | **🟑** | 7 |
| Roesel | 2022 | **🟑** | **🟑** | **🟑** | **🟑** | **🟑** | **🟑** | **🟑** | / | 7 |
| Schou | 2017 | **🟑** | **🟑 ** | **🟑** | / | / | **🟑** | / | **🟑** | 5 |
| Sclafani | 2018 | **🟑** | **🟑** | / | **🟑** | **🟑** | **🟑** | **🟑** | / | 6 |
| Sun | 2014 | **🟑** | **🟑** | **🟑** | / | **🟑** | **🟑** | **🟑** | **🟑** | 7 |
| Tie | 2018 | **🟑** | **🟑** | **🟑** | **🟑** | **🟑** | **🟑** | / | **🟑** | 7 |
| Truelsen | 2022 | **🟑** | **🟑 ** | **🟑** | **🟑** | / | **🟑** | **🟑** | **🟑** | 7 |
| Vidal | 2021 | **🟑** | **🟑** | **🟑** | **🟑** | **🟑** | / | **🟑** | / | 6 |
| Wang | 2021 | **🟑** | **🟑** | / | / | **🟑** | **🟑** | **🟑** | **🟑** | 6 |
| Zhou | 2020 | **🟑** | **🟑** | **🟑** | **🟑** | / | **🟑** | **🟑** | **🟑** | 7 |
| Zitt | 2008 | **🟑** | **🟑** | / | / | **🟑** | **🟑** | **🟑** | **🟑** | 6 |

| **Name** | **Year** | **Country** | **Journal** | **Impact Factor** |
| --- | --- | --- | --- | --- |
| Agostini | 2011 | Italy | Annals of Surgical Oncology | 5.3 |
| Alden | 2024 | USA | The Oncologist | 5.8 |
| Appelt | 2020 | UK | American Journal of Clinical Oncology | 2.6 |
| Boysen | 2017 | Denmark | Tumor Biology | 3.7 |
| Guo | 2020 | China | Clinical and Translational Medicine | 10.6 |
| Hofste | 2023 | Netherlands | EJSO | 4 |
| Khakoo | 2019 | UK | Clinical Cancer Research | 11.5 |
| Liu | 2022 | China | eBioMedicine | 11.2 |
| McDuff | 2021 | USA | JCO Precision Oncology | 4.6 |
| Morais | 2023 | Portugal | MDPI Pharmaceuticals | 4.6 |
| Murahashi | 2020 | Japan | British Journal of Cancer | 8.8 |
| Pazdirek | 2020 | Czech Republic | Frontiers in Oncology | 4.7 |
| Roesel | 2022 | Switzerland | Frontiers in Oncology | 4.7 |
| Schou | 2017 | Denmark | Annals of Oncology | 50.5 |
| Sclafani | 2018 | UK | Scientific Reports | 4.6 |
| Sun | 2014 | Chna | Oncology Reports | 3.9 |
| Tie | 2018 | Australia | BMJ | 107.7 |
| Truelsen | 2022 | Denmark | Clinical and translational radiation oncology | 3.1 |
| Vidal | 2021 | Spain | Clinical Cancer Research | 11.5 |
| Wang | 2021 | China | PLOS Medicine | 11.6 |
| Zhou | 2020 | China | Clinical Cancer Research | 11.5 |
| Zitt | 2008 | Austria | Disease Markers | 3.5 |

**Supplementary material S3: Methodological characteristics of included studies**
